# Ovarian-derived signals reprogram hepatic lipid metabolism in aging: Implications for therapeutic targeting of metabolic dysfunction

**DOI:** 10.1556/1661.2026.00112

**Published:** 2026-05-08

**Authors:** Nathan D. McCoy, Michael T. Kinter, Augusto Schneider, Fabiola Santos, Michal M. Masternak, Jeffrey B. Mason

**Affiliations:** 1 College of Veterinary Medicine, Department of Veterinary Clinical and Life Sciences, Center for Integrated BioSystems, Utah State University, Logan, UT, 84322, USA; 2 Aging and Metabolism Research Program, Oklahoma Medical Research Foundation, Oklahoma City, Oklahoma, USA; 3 Faculdade de Nutrição, Universidade Federal de Pelotas, Pelotas-RS, Brasil; 4 College of Medicine, Burnett School of Biomedical Sciences, University of Central Florida, Orlando, FL, USA; 5 Department of Head and Neck Surgery, Poznan University of Medical Sciences, Poznan, Poland

**Keywords:** menopause, ovarian transplantation, aging, *β*-oxidation, hepatic metabolism, triglycerides, lipid metabolism, metabolic reprogramming, pharmacological mechanisms, PPARα signaling

## Abstract

**Background and Aim:**

Aging in mammals is a complex, multifaceted process involving the progressive decline of physiological functions, in which energy metabolism plays a pivotal role. *β*-oxidation—the primary pathway for converting fatty acids into energy—is tightly linked to aging and to female reproductive senescence. In the present study, we investigated how ovarian tissue remodels hepatic proteins central to the *β*-oxidation pathway.

**Methods:**

Liver proteomes were analyzed in CBA/J control mice at 4, 13, 23, and 27 months of age and compared with those of 23-month-old mice that received transplants of young ovarian tissue at 13 months. *β*-oxidation proteins were quantified using high-resolution mass spectrometry. Serum triglycerides were measured enzymatically, and metabolic cage analyses were performed to assess substrate utilization and energy balance.

**Results:**

Ovarian tissue transplantation substantially modulates the expression of *β*-oxidation-related proteins and reduces systemic lipid accumulation in aged mice. These proteomic shifts are consistent with enhanced metabolic efficiency and decreased oxidative stress—mechanisms well-established as drivers of extended health span and longevity. Results apply to menopause dyslipidemia, insulin resistance, metabolic syndrome, NAFLD, and aging. Transplants reduce *β*-oxidation proteins and triglycerides, improving hepatic lipid clearance and steatosis risk. Identifying ovarian signals enables non-surgical mimics via peptides or modulators. Changes align with PPARα agonists, CPT1 modulators, mitochondria-targeted antioxidants (MitoQ), and NAD^+^/sirtuin pathways—offering testable routes to replicate ovarian benefits.

**Conclusions:**

Ovarian-derived signals induce metabolic reprogramming in aged mice, characterized by reduced *β*-oxidation protein expression, decreased triglyceride accumulation, and improved metabolic efficiency. These findings provide a molecular framework for understanding how ovarian-derived factors may govern metabolism and organismal health during aging, with potential implications for interventions targeting age-related metabolic decline.

## INTRODUCTION

Fatty acids are a major energy source in mammals and, when metabolized through *β*-oxidation, generate ATP, supporting energy-intensive processes in the heart, skeletal muscles, kidneys, and other organs [1]. Beyond energy production, *β*-oxidation influences lipid accumulation, peroxidation, and trafficking, contributing to cellular homeostasis [2]. When lipid metabolism is repressed, excess lipid accumulation can promote insulin resistance, inflammation, and neurodegenerative diseases [3, 4].

Fatty acids are classified as saturated (SFAs; no double bonds), monounsaturated (MUFAs; one double bond), or polyunsaturated (PUFAs; multiple double bonds), with triglycerides serving as their primary storage form in the body [5]. Accumulation of SFAs promotes intracellular inflammation [4], whereas excess MUFAs and PUFAs are highly susceptible to attack by reactive oxygen species (ROS), resulting in lipid peroxidation and the generation of toxic intermediates that damage cellular membranes [4].

Premenopausal women typically enjoy a health advantage over age-matched men, but this benefit is lost or attenuated in surgically menopausal women and in those with premature ovarian insufficiency [6–9]. In this context, sex differences in lipid metabolism become especially pronounced with advancing age and play a central role in the maintenance of health. Postmenopausal women undergo more extensive lipidomic changes than men of comparable age, directly elevating their risk of metabolic and neurodegenerative diseases, including Alzheimer’s disease. Notably, centenarian women display a distinctive shift in the PUFA-to-MUFA ratio that is absent in centenarian men, establishing that age-related remodeling of lipid metabolism is both tissue-specific and sex-dependent [5]. In ovariectomized rodent models, *β*-oxidation and other fatty acid oxidation pathways are significantly impaired, an effect commonly attributed to the loss of ovarian function and circulating estradiol [10–14]. Consistent with these preclinical findings, surgically menopausal women exhibit higher triglyceride levels and lower HDL-cholesterol compared with women who undergo natural menopause and retain their senescent ovaries [15]. Together, these observations establish that ovarian tissue is an important regulator of systemic metabolic homeostasis.

Previous work from our laboratory has shown that transplantation of young ovaries into post-reproductive female mice extends overall health span and lifespan, an effect that is independent of ovarian germ cells and cyclical estradiol production [16–24].

Ovarian-derived factors further influence lipid transport and brain health by modulating apolipoprotein expression [25–28]. In post-reproductive mice, ovarian transplantation restores apolipoprotein profiles to levels observed in young females, reduces neuroinflammation (gliosis), and improves cognitive performance [29, 30]. These results establish a direct functional connection between ovarian signaling, lipid regulation, and systemic metabolic health.

Despite these insights, the mechanistic links among ovarian function, *β*-oxidation, and organismal aging remain largely unexplored. We therefore provide evidence that transplantation of young ovarian somatic cells modulates *β*-oxidation and lipid metabolism in post-reproductive female mice, suggesting a molecular pathway by which ovarian-derived factors may contribute to health span and longevity.

## MATERIALS AND METHODS

### Animals

CBA/J female mice, a well-established model for post-reproductive aging due to early ovarian follicle loss [17, 19, 20], were obtained from the National Institute on Aging and Jackson Laboratory. Mice were housed individually in ventilated cages under controlled temperature (21 ± 2 °C), humidity (50 ± 20%), and a 12:12 h light-dark cycle, with ad libitum access to chow and water. Environmental enrichment and exposure to male bedding were provided throughout the study to simulate natural social cues. All procedures were approved by the Utah State University IACUC (#10222) and conducted in an AAALAC-accredited facility according to NIH guidelines.

### Surgical procedures

Surgical procedures, anesthesia, and post-operative analgesia were administered as previously described [18]. Mice were monitored at least twice daily, with humane endpoints established in consultation with the attending veterinarian. Euthanasia was performed by cervical dislocation followed by thoracotomy and exsanguination.

### Experimental design

**Fig. 1.**
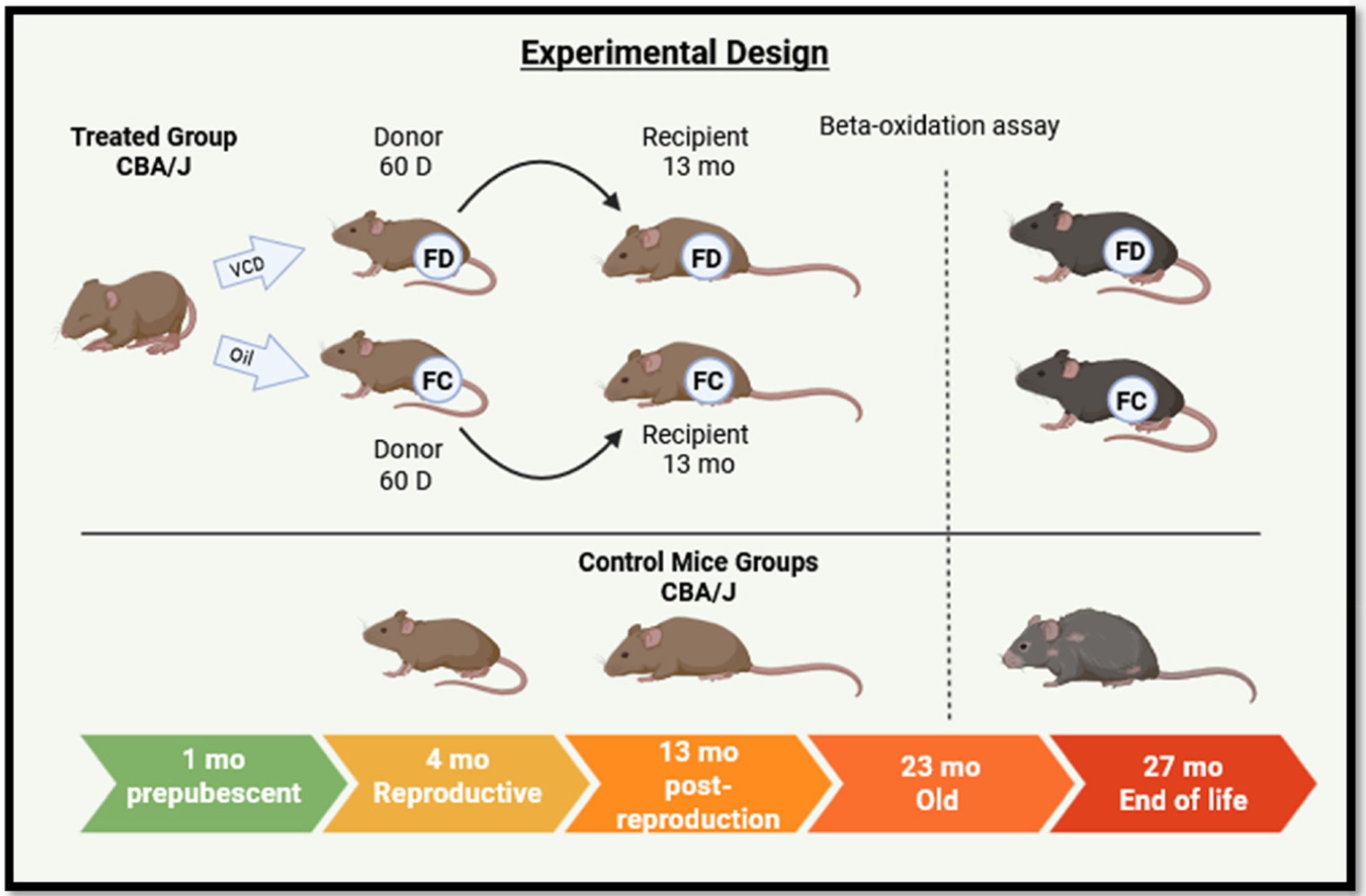
Experimental design. Donor mice were treated with 4-vinylcyclohexene diepoxide (VCD) or vehicle (sesame oil) for 20 days. Ovarian tissues were collected from 60-day-old donor mice and transplanted into 13-month-old recipient mice following bilateral ovariectomy. Control mice were analyzed for *β*-oxidation protein expression at 4 (*n* = 5), 13 (*n* = 2), and 27 (*n* = 3) months of age. Recipient mice were analyzed at 23 months of age. Two types of ovarian grafts were used: follicle-containing (FC; *n* = 3) and follicle-depleted (FD; *n* = 6). Follicle depletion was achieved by VCD treatment, which selectively eliminates primordial and primary follicles while preserving ovarian somatic tissue. Vehicle-treated donor mice (OIL) received sesame oil only Abbreviations: VCD, 4-vinylcyclohexene diepoxide; FC, follicle-containing ovarian tissue; FD, follicle-depleted ovarian tissue; OIL, vehicle-treated controls. Created with BioRender.com

Experimental design and grouping followed previously described paradigms [30]. Briefly, reproductively senescent (13-month-old) CBA/J females received ovarian tissue transplants from 60-day-old donors (Fig. 1). Control mice retained their endogenous ovaries and were not surgically manipulated, as prior work demonstrated no physiological differences between intact and sham-operated animals [18–21].

### Ovarian transplantation

Ovarian transplantation procedures were conducted as previously described [18, 20, 31]. In brief, 13-month-old recipient mice underwent bilateral ovariectomy followed by transplantation of germ cell/follicle-containing (FC/GC) or germ cell/follicle-depleted (FD) ovarian tissue from 60-day-old donors of the same strain. Donor and recipient mice were anesthetized via intraperitoneal injection (ketamine, xylazine, acepromazine cocktail), and donor ovaries were excised and transferred into the recipient ovarian bursa. The incision was sutured and closed with wound clips. Post-operative mortality was <5%.

### Follicle (germ cell) depletion

Follicle depletion was achieved using 4-vinylcyclohexene diepoxide (VCD) as previously described [29, 32]. Briefly, 28-day-old CBA/J females received daily intraperitoneal injections of 160 mg kg^−1^ VCD in sesame oil for 20 days. Vehicle-treated (oil-only) mice served as controls. At 60 days of age, the ovaries were collected for transplantation. VCD treatment effectively depleted primordial and primary follicles while leaving somatic tissue intact, as verified by histological assessment [16].

### β-Oxidation protein assay

Liver samples were collected at euthanasia, flash frozen, and processed for proteomic *β*-oxidation analysis. Pulverized liver tissue (50–200 mg) was reconstituted in RIPA buffer, heated (70 °C, 15 min), and clarified by centrifugation. Supernatants were aliquoted, normalized to 100 μg total protein, and analyzed by high-resolution accurate-mass (HRAM) spectrometry (Thermo QExactive Plus) as previously validated by Dr. Kinter (Aging and Metabolism Research Program, Oklahoma Medical Research Foundation). Proteins were digested with trypsin, and peptides were analyzed by LC–MS/MS. Quantification was performed using Skyline software with BSA as an internal standard. Each protein was quantified from the geometric mean of two validated peptides and expressed as pmol/100 μg total protein.

### Triglyceride assay

Serum triglyceride concentrations were measured using the Infinity Triglycerides Reagent (Thermo Fisher, TR22321) and Triglyceride Standard (Pointe Scientific, T7531-STD) according to the manufacturer’s instructions, with minor modifications. Briefly, 10 μL of each serum sample or triglyceride standard dilution was transferred in duplicate into a 96-well microtiter plate. Subsequently, 190 μL of Triglycerides Reagent was added to each well and gently mixed using a multichannel pipette to avoid bubble formation. Plates were incubated at 37 °C for 5 min, and absorbance was measured at 500 nm using a Tecan Infinite M200 plate reader.

If sample triglyceride concentrations exceeded 200 mg dL^−1^, the serum was diluted with phosphate-buffered saline (PBS) to maintain a total volume of 200 μL per well, and concentrations were adjusted using the corresponding dilution factor.

### Metabolic cage assay

Indirect calorimetry measurements were obtained using a comprehensive metabolic monitoring system (Columbus Instruments, CLAMS) following the manufacturer’s guidelines, with minor modifications. Briefly, each mouse was singly housed in a ventilated metabolic cage, and the system was calibrated for O_2_ and CO_2_ analyzers before animal loading. Following a 24-h acclimation period, VO_2_, VCO_2_, respiratory quotient (RQ), heat production, food intake, water intake, and ambulatory activity were recorded at 10-min intervals over a 48-h monitoring period. Environmental parameters were maintained at 22 °C with a 12-h light/dark cycle.

### Statistical analysis

Data were analyzed using GraphPad Prism 7.04. Normality was assessed via the D’Agostino–Pearson omnibus test. Group comparisons were made using two-way ANOVA with Tukey–Kramer post hoc tests or unpaired two-tailed Student’s *t*-tests assuming unequal variance. Significance was set at *P* < 0.05. Mean standard error (SE) for percent change in pmoL protein was 1.6%; changes below this threshold were considered not significant (NC).

Due to COVID-19–related facility restrictions, 23-month-old control mice were unavailable at the time of the experimental endpoint. To enable comparison, theoretical 23-month control values for each *β*-oxidation protein were estimated by curve fitting across the measured values at 4, 13, and 27 months of age. We acknowledge that this is a methodological limitation: curve-fitted values cannot capture cohort-specific variation, and statistical comparisons against theoretical controls should be interpreted with appropriate caution. This limitation is reflected in the framing of results as “significantly different from theoretical control values” rather than from empirically measured controls. Replication with prospectively collected age-matched controls will be necessary to fully validate these findings.

## RESULTS

### β-oxidation proteins

**Fig. 2.**
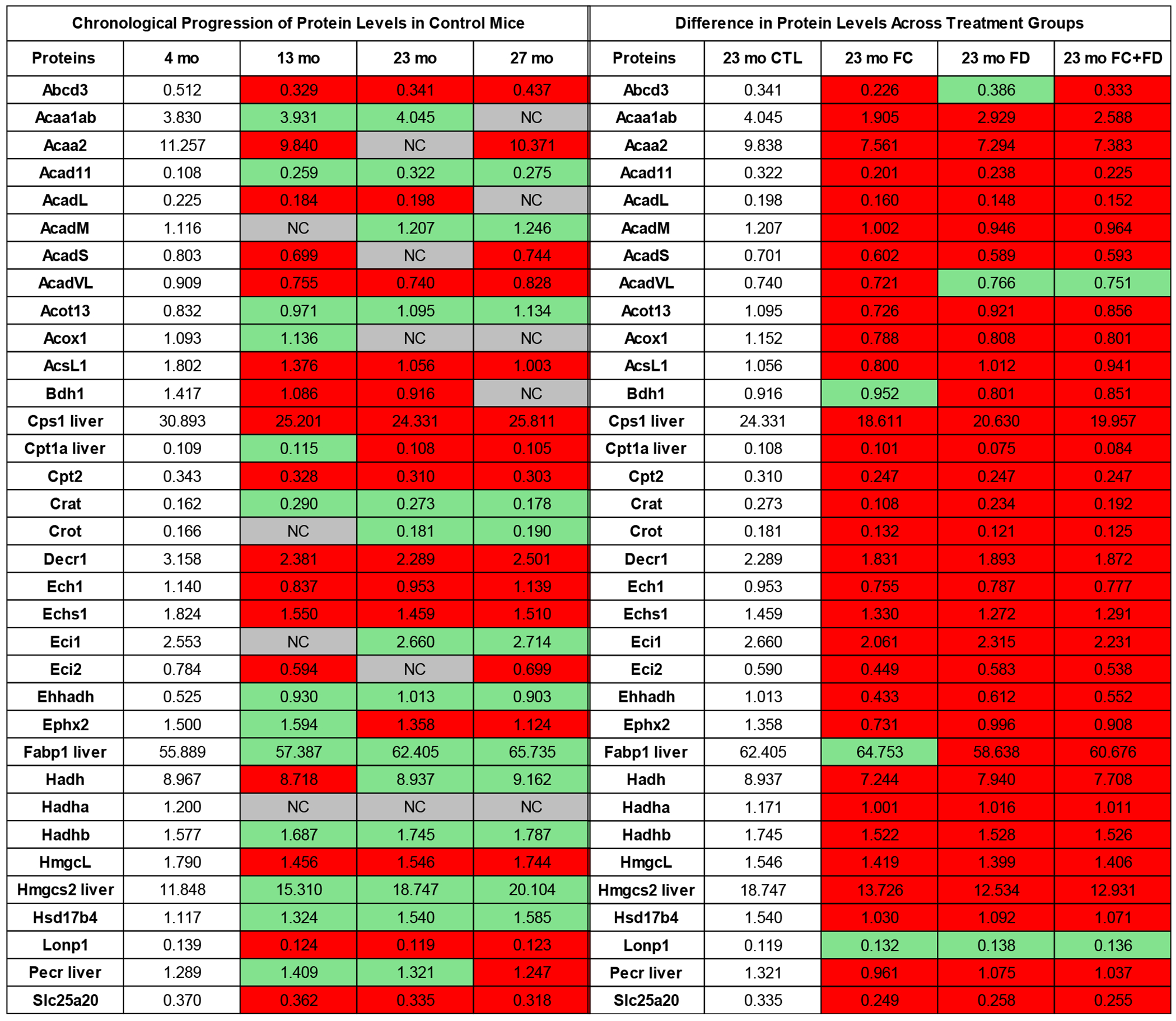
Heatmap of β-oxidation protein expression across age and treatment groups. The heatmap displays the percentage change in protein expression between control mice at 4, 13, 23, and 27 months of age (reflecting chronological changes) and between treatment groups at 23 months of age (reflecting experimental effects of ovarian transplantation). Treatment groups include follicle-containing (FC) and follicle-depleted (FD) ovarian transplant recipients. Green indicates increased protein expression, red indicates decreased protein expression, and values with changes ≤1.6% are designated as NC (no change)

β-oxidation proteins were measured at different life stages (Fig. 2) in control mice (4, 13, 23, 27 months) that represent the different states of reproduction in mice which included the young, healthy and reproductively active mice at 4 months of age, mice that had recently undergone reproductive failure/complete cessation of reproductive cycling at 13 months of age (most mice experience reproductive failure/complete cessation of reproductive cycling by approximately 12 months of age in this strain), old mice at 23 months, and mice at 27 months that have gone past expected life span.

The median lifespan of CBA/J mice is approximately 661 days (~22 months) [18]. Because inclusion in post-reproductive groups required survival to 13 months (~395 days), mice that died prior to this time point were excluded, resulting in a shift in observed mean lifespan of approximately 726 days. 23- and 27-month control groups represent advanced- and extreme-aged survivors relative to the expected lifespan of CBA/J mice.

**Fig. 3.**
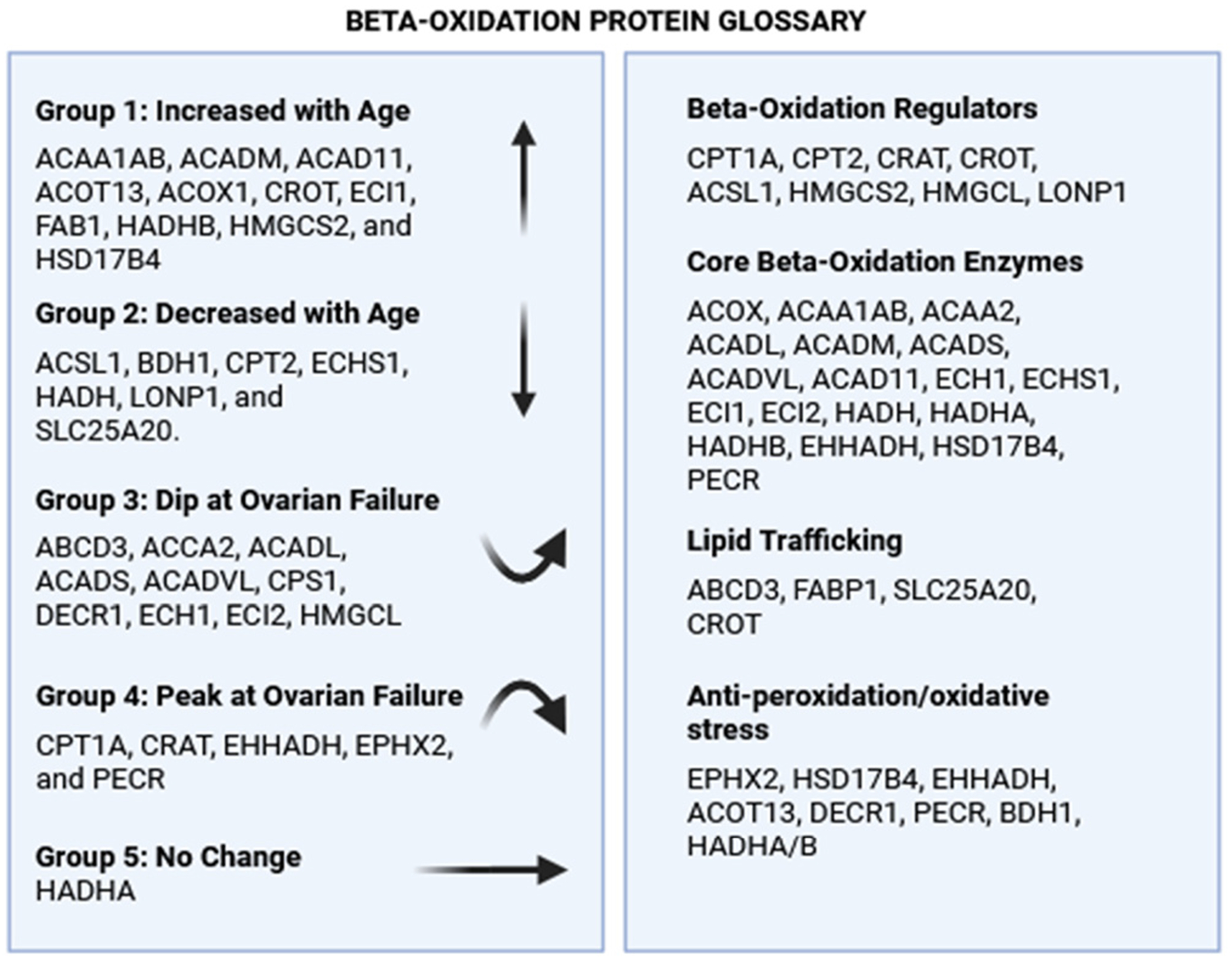
β-oxidation protein glossary. This figure summarizes the general trends in *β*-oxidation protein expression across control mice at 4, 13, 23, and 27 months of age and highlights the primary functional roles of each protein within fatty acid metabolism. Some proteins have multiple functions and are included in more than one functional category. This schematic is intended to provide a visual framework for interpreting age-associated changes in *β*-oxidation pathways

**Fig. 4.**
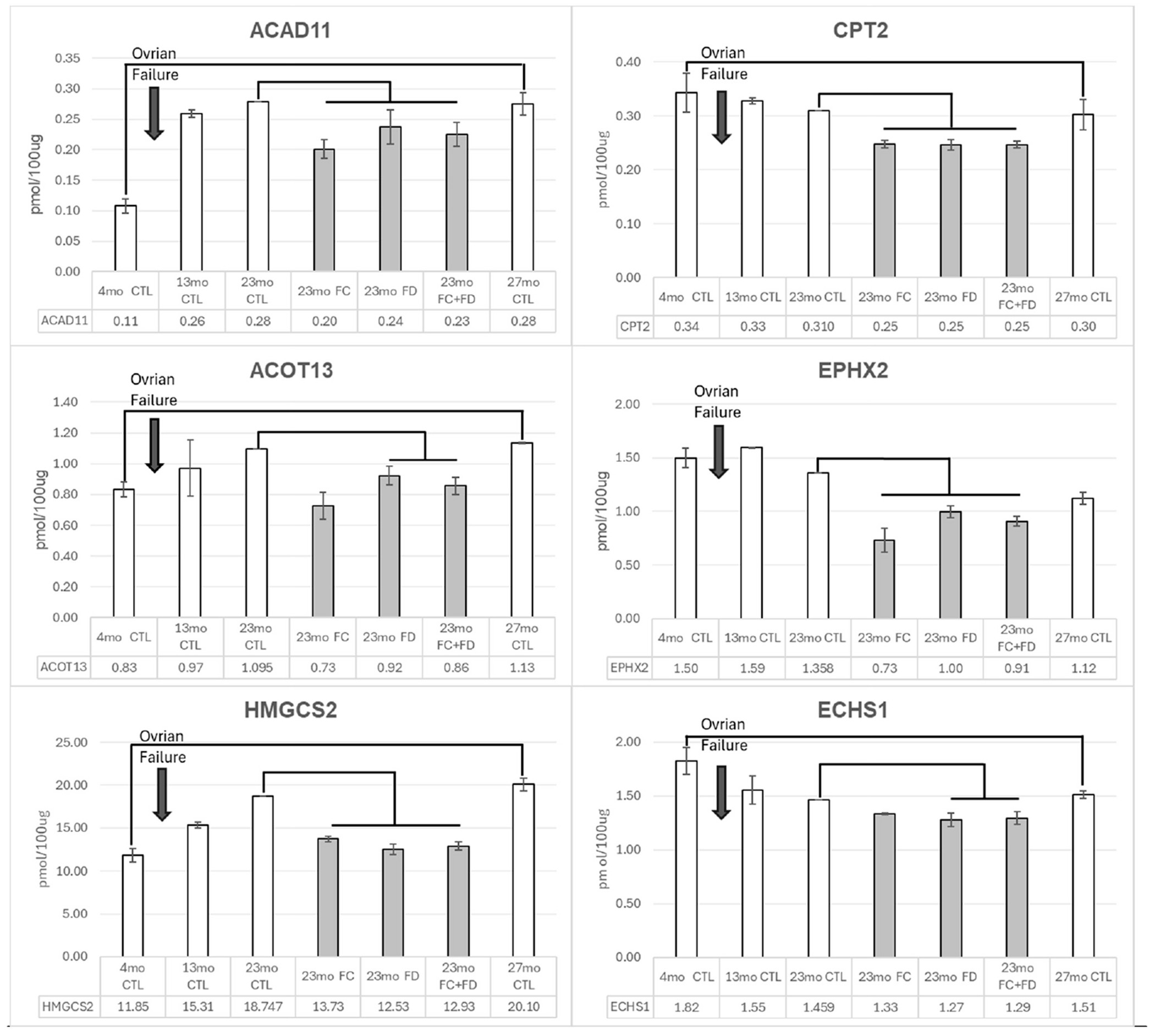
Examples of β-oxidation proteins exhibiting age-related changes and responses to ovarian transplantation. Acyl-CoA dehydrogenase family member 11 (ACAD11), acyl-CoA thioesterase 13 (ACOT13), and 3-hydroxy-3-methylglutaryl-CoA synthase 2 (HMGCS2) increased in expression with age and decreased following transplantation of young ovarian tissue. In contrast, enoyl-CoA hydratase, short chain 1 (ECHS1) and carnitine palmitoyltransferase 2 (CPT2) decreased with age and were further reduced in transplant recipients. Epoxide hydrolase 2 (EPHX2) exhibited an initial increase at the time of ovarian failure, followed by a decline with advancing age, and was further decreased following ovarian transplantation

β-oxidation proteins in Groups 1 and 2 exhibited protein-specific age-associated increases and decreases across the three control groups, reflecting a model of age-related change (Fig. 3). Many of the proteins exhibited the greatest change in protein levels between 4 and 13 months (Fig. 4). Due to sample size constraints (4-month CTL *n* = 5, 13-month CTL *n* = 2, 27-month CTL *n* = 3) only two proteins showed statistically significant differences between 4 and 13 months (ACADVL and ECI1); visual trends reveal broad changes in protein expression during reproductive failure, with Groups 3 and 4 showing the greatest degree of change between 4 and 13 months.

This pattern demonstrates that reproductive status is a major factor governing *β*-oxidation protein expression. In contrast, Groups 1 and 2 (23-month and 27-month controls) exhibited a reversal in the direction of several protein changes, confirming that these alterations are not sustained and undergo compensation across late life.

β-oxidation proteins were measured in FC and FD recipient mice at 23 months of age (transplantation occurred at 13 months of age). These values were then compared to those of 23-month-old control mice. In control mice, *β*-oxidation proteins changed with age, with a notable distinction observed between beginning (4 months) and end (27 months) of the study (Fig. 4). Age-matched (23-month) control values were then used to determine if the transplantation of FC/FD ovarian somatic tissues caused the experimental values of the *β*-oxidation proteins to deviate significantly from the trendline established by the 4 control groups.

Transplantation of young FC ovaries decreased protein levels in 31 out of 34 (92.2%) *β*-oxidation proteins observed compared to 23-month-old control values. Proteins BDH1, FAB1 Liver, and LONP1 increased in protein level with transplantation. 22 of 34 (64.7%) proteins were significantly different than the theoretical control value. Transplantation of young FD ovaries decreased protein levels in 32 out of the 34 proteins observed compared to the estimated 23-month control value; proteins ABCD3 and LONP1 increased with young FD ovary transplantation. In FD mice, 27 out of 34 (79.4%) proteins were significantly different than the theoretical control value. When combined, the transplant groups showed decreased protein levels for 33 of the 34 proteins, with LONP1, a regulatory protein, the exception. 28 out of 34 (82.4%) were significantly different than the theoretical control value.

### Physiological and metabolic outcomes

#### Triglycerides.

**Fig. 5.**
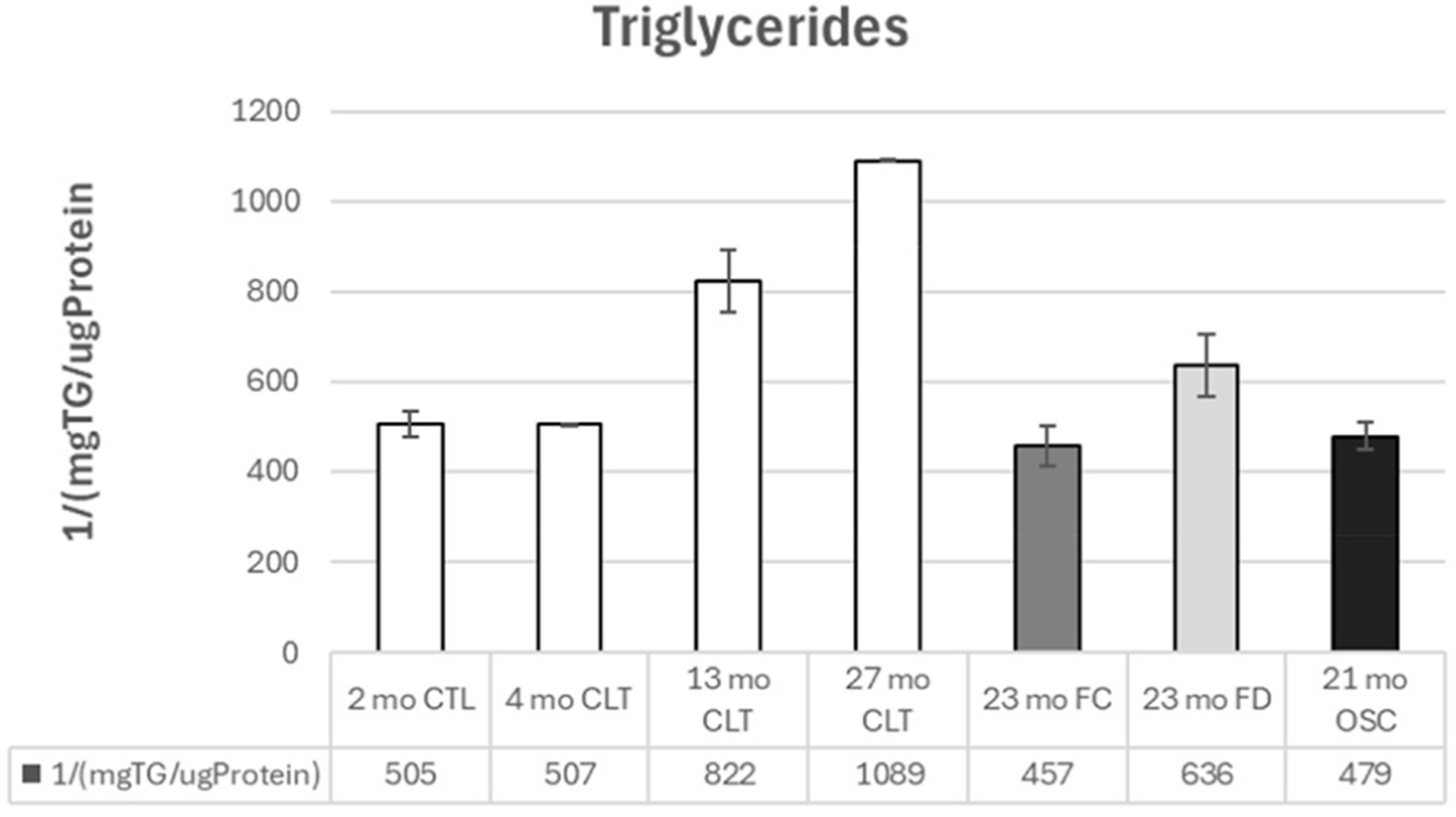
Triglyceride levels in CBA/J female mice. Circulating triglyceride levels increased with age in control mice and were reduced following transplantation of young ovarian tissue. Treatment groups included follicle-containing ovarian grafts (FC), follicle-depleted ovarian grafts (FD), and ovarian somatic cell transplants (OSC) Abbreviations: FC, follicle-containing ovarian tissue; FD, follicle-depleted ovarian tissue; OSC, ovarian somatic cells

Triglyceride levels increased progressively with age in our CBA/J controls (Fig. 5), reaching peak concentrations in 27-month-old control mice. Both FC and FD recipient mice exhibited dramatically lower circulating triglyceride levels compared to age-matched controls, effectively restoring a lipid profile characteristic of young, reproductively active mice.

#### Metabolic cage analysis.

Pilot metabolic cage data were collected from CB6F1/J mice (Table 1), which are a genetically distinct strain from the CBA/J mice used for all other experiments in this study. These data are therefore presented as preliminary and cross-strain findings; they may not directly reflect the metabolic responses of CBA/J mice and should be interpreted accordingly. CB6F1/J mice that underwent the same ovarian treatments were used to collect pilot data in metabolic cages. In these pilot assays, ovarian somatic tissue was transplanted directly into the ovarian bursa. Recipients exhibited a consistently lower respiratory quotient (${\text{RQ}=\dot{\text{V}}\text{CO}}_{2}/{\dot{\text{V}}\text{O}}_{2}$) compared to control mice (0.75–0.79, respectively). RQ provides an estimate of substrate utilization, with values closer to 0.7 indicating greater fatty acid oxidation and values closer to 1.0 indicating carbohydrate oxidation [33]. All animals were maintained on a standard laboratory chow (LabDiet 5001, PMI Nutrition International, St. Louis, MO), which provides approximately 3.3 kcal g^−1^ and derives ~28% of calories from protein, ~13–14% from fat, and ~58–60% from carbohydrates.

#### Weight and feed intake of CBA/J mice.

Body weight and feed intake were tracked over a 49-day monitoring period across all groups.

Baseline body weights were comparable among groups, ranging from 24.86 to 27.73 g (Table 2). By the end of the study, all groups exhibited a reduction in body weight. The 13-month control group decreased from 27.67 to 26.54 g (−1.13 g), while the 27-month control group showed the greatest decline, decreasing from 24.86 to 21.54 g (−3.32 g). The FC group maintained body weight over the study period (26.32 to 26.31 g; −0.01 g), whereas the FD group exhibited a modest reduction (27.73 to 26.65 g; −1.08 g).

Average feed intake differed among groups. The FC group demonstrated the highest average intake (40.67 g per week), followed by the FD group (37.18 g), the 27-month control group (32.21 g), and the 13-month control group (28.88 g). Despite higher feed consumption in the FC and FD groups, these mice did not exhibit proportional increases in body weight over the monitoring period.

## DISCUSSION

We previously demonstrated that the transplantation of young ovarian somatic tissues or cells enhances the health of post-reproductive recipient mice, independent of follicles and cyclic estrogens [17, 24, 29, 30]. In the current experiment, we establish that control mice showed the most significant changes in *β*-oxidation protein levels between 4 and 13 months of age, confirming that ovarian failure is a key driver of decreased expression of these proteins. Recipients of follicle-containing (FC) and follicle-depleted (FD) ovarian tissues displayed notably lower levels of *β*-oxidation proteins compared to controls, with 82% of these proteins reduced in recipient mice relative to control groups. Consistent with observations in women, triglyceride levels increased with age in our CBA/J female mice but declined sharply in the transplanted groups, returning to levels that reflect the biological age of the ovarian tissue rather than the chronological age of the recipient [15]. These findings demonstrate that ovarian tissue transplantation effectively reverses age-related accumulation of circulating triglycerides (Fig. 5), and that concurrent reductions in both *β*-oxidation proteins and triglyceride levels reveal a broad metabolic recalibration—not merely increased *β*-oxidation flux—in transplant recipients [34].

β-oxidation proteins exhibited diverse functions and presented various trends across aging and transplantation. For instance, ACAD11, ACOT13, and HMGCS2 increased with age in control mice, while ECHS1, EPHX2, and CPT2 decreased. All six proteins were expressed at lower levels in FC/FD recipients compared to 23-month-old controls. ACAD11 is involved in processing hydroxylated fatty acids in peroxisomes and very-long-chain fatty acid *β*-oxidation [35, 36]. ACOT13 hydrolyzes medium- and long-chain acyl-CoAs into free fatty acids, acting as a regulatory “brake” on *β*-oxidation to maintain energy balance [37, 38]. HMGCS2 plays a crucial role in mitochondrial regulation of ketogenesis and fatty acid oxidation [39, 40]. The increased expression of these enzymes with age in control mice indicates a rising need for regulatory compensation due to accumulating metabolic dysfunction. Strikingly, FC/FD recipients exhibited expression patterns indistinguishable from those of 4-month controls, demonstrating that the metabolic environment of 23-month-old FC/FD mice has undergone comprehensive metabolic reprogramming toward a more youthful metabolic profile.

The decline of age-related proteins also provides valuable insight. ECHS1 is essential for short-chain fatty acid *β*-oxidation [41, 42]. EPHX2 metabolizes fatty acid epoxides, protecting against lipid peroxidation and neurological dysfunction [43]. CPT2 converts long-chain acyl-carnitines into acyl-CoAs within mitochondria, essential for effective fatty acid catabolism; its dysfunction leads to hepatic steatosis [44]. The reduction of these proteins with age reflects progressive impairment of mitochondrial and peroxisomal fatty acid processing. FC/FD recipients also exhibited low levels of these proteins, demonstrating that ovarian-derived signals eliminate the need for these age-associated compensatory pathways—a hallmark of restored metabolic health.

The overall decrease in *β*-oxidation proteins in FC and FD groups reflects metabolic recalibration rather than direct suppression of fatty acid oxidation, consistent with the substantially increased feed intake observed in recipient mice [45]. This interpretation warrants explicit discussion, as the relationship between reduced *β*-oxidation protein levels and improved metabolic health is not immediately intuitive. In metabolically stressed or aged animals, the paradoxical upregulation of fatty acid oxidation enzymes reflects chronic overreliance on lipid as a primary fuel source —a hallmark of impaired metabolic flexibility in which diminished glucose uptake and insulin resistance force the cell to compensate by maximizing *β*-oxidation capacity. Conversely, the reduction in *β*-oxidation enzyme abundance observed in transplant recipients is consistent with restored substrate switching—a shift from predominant lipid utilization toward a more flexible metabolic state capable of efficiently oxidizing glucose. In this context, lower *β*-oxidation protein expression reflects reduced reliance on fatty acid oxidation under conditions of improved metabolic competence, rather than impaired lipid catabolism. This interpretation is supported by the concurrent sharp decline in circulating triglycerides, the restoration of glucose tolerance reported in prior work from this laboratory [24], and the lower respiratory quotient observed in metabolic cage experiments, all of which are consistent with a coordinated shift in substrate preference rather than simple enzyme loss.

The metabolic phenotype of FC/FD recipients closely resembles that of caloric restriction (CR) models, which are likewise characterized by improved metabolic efficiency, reduced hepatic lipid burden, and enhanced mitochondrial function—without necessarily displaying elevated *β*-oxidation flux. The CR-like state is one of improved efficiency rather than increased throughput: less lipid must be oxidized because less accumulates and because glucose is utilized more effectively. The sharp decline in triglyceride levels among FC/FD recipient mice, without any dietary restrictions, confirms a fundamental shift in systemic energy metabolism. Our prior research has demonstrated that manipulation of ovarian function powerfully influences metabolism, as evidenced by markedly increased glucose tolerance following the transplantation of young ovarian somatic tissues into aged CBA/J females [24]. These findings, together with the estradiol-independent effects of ovarian somatic tissues on inflammation, motor function, and body composition [16, 19, 23, 29], confirm that young ovarian tissue exerts potent, hormone-independent effects on whole-body metabolic homeostasis. This is further validated by our pilot data from metabolic cages, which showed decreased O2/CO2 consumption alongside a striking 100% increase in feed consumption and general activity among FC/FD recipients, demonstrating improved metabolic efficiency that closely parallels findings in established longevity mouse models such as GHRH-KO mice [46].

The metabolic flexibility of FC/FD mice was restored to that of young 4-month-old mice; reliance on fatty acid oxidation is diminished, and fatty acid synthesis is reduced. This restoration is driven by ovarian-derived factors that enhance glucose uptake through restored insulin sensitivity, thereby suppressing de novo lipogenesis (DNL) in the liver [47, 48]. The decrease in key *β*-oxidation regulators, including CROT, CPT1A, and HMGCS2, with age in control mice reflects progressive overreliance on fatty acid oxidation as metabolic flexibility deteriorates. In FC/FD recipients, their reduced expression reflects a fundamentally healthier metabolic environment characterized by lower circulating fatty acids and diminished demand for sustained *β*-oxidation. This metabolic profile parallels aspects of caloric restriction (CR), which is characterized by reduced hepatic lipid burden, improved insulin sensitivity, and enhanced metabolic efficiency rather than increased fatty acid flux [49–51]. Critically, CR is referenced here as a comparative metabolic state, not as a direct mechanistic equivalent to transplantation. Indeed, despite the substantially increased feed intake observed in FC/FD recipients, their metabolic phenotypes recapitulate key features of CR models, including improved metabolic efficiency and reduced circulating lipid levels. These improvements occur in the context of elevated caloric intake, providing unambiguous evidence that ovarian-derived factors actively enhance nutrient utilization efficiency and optimize whole-body metabolism—effects entirely independent of caloric restriction.

The reduction of *β*-oxidation proteins in transplant recipients reflects enhanced metabolic efficiency, antioxidant function, and lipid transport. Proteins such as EPHX2, DECR1, and BDH1, associated lipid metabolism and anti-peroxidation properties, decreased with age in control mice and further declined in FC/FD recipients. Consistent with prior work from our laboratory, ovarian transplantation robustly reduces oxidative stress [29, 52]. Reduced oxidative stress indicates the efficiency of *β*-oxidation is enhanced, preventing the formation of damaging oxidative intermediates [4, 53]. This pattern closely mirrors that observed in long-lived communities such as the Okinawans, whose diets rich in omega-3s and omega-6s yield lower rates of neurodegenerative diseases and reduced lipid peroxidation [4, 5, 54, 55]. These long-lived populations exhibit resilience to peroxidation, maintaining regulatory factors that optimize fatty acid oxidation efficiency—a phenotype now recapitulated in FC/FD recipient mice through ovarian tissue transplantation. Collectively, these findings are consistent with the interpretation that ovarian tissue contributes to a broad recalibration of energy metabolism and an enhancement of cellular resilience through improved oxidative balance.

The proteomic data provide evidence consistent with the conclusion that exposure to young ovarian tissue induces profound metabolic remodeling in aged mice. The observed shifts demonstrate improved substrate flexibility and mitochondrial efficiency, consistent with metabolic reprogramming toward a more youthful metabolic profile and the mechanisms underlying longevity. Future studies will build on these findings through direct metabolic assays (respiration and flux analyses) alongside comprehensive lipidomic and pathway profiling (AMPK, PPARα, IGF, and mitochondrial biogenesis markers). Such investigations will further characterize whether these proteomic changes represent true metabolic reprogramming or a more targeted reorganization of substrate utilization, ultimately identifying the specific molecular mediators by which ovarian-derived factors promote systemic metabolic health and extended lifespan. Together, these findings demonstrate that ovarian-derived signals reprogram hepatic lipid metabolism, shifting aged animals toward a more metabolically efficient and youthful state and establishing ovarian signaling as a regulator of systemic metabolic aging and a promising target for therapeutic intervention.

### Limitations

These limitations should be considered when interpreting these findings. First, due to COVID-19–related facility closures, no direct 23-month-old control mice were available. Age-matched control values for *β*-oxidation proteins were therefore estimated by curve fitting from data at 4, 13, and 27 months. Second, post-reproductive groups included only mice surviving to 13 months, introducing survival-selection bias. This elevated mean lifespan (~726 days vs. strain median ~661 days) and limits generalizability to the broader CBA/J population. Third, metabolic cage data were obtained from CB6F1/J mice, not CBA/J. Strain differences in genetics, body composition, and baseline metabolism mean these results are preliminary and cross-strain in nature. Finally, a causal link between the observed proteomic changes and the lifespan/healthspan benefits of ovarian transplantation remains unproven. Identifying specific ovarian-derived factors and their targets will require further targeted investigation.

### Translational perspective

These findings have translational relevance across several clinical contexts: menopause-associated dyslipidemia and insulin resistance, metabolic syndrome, NAFLD, and broader aging and longevity research. The proteomic signature in transplant recipients—reduced *β*-oxidation protein expression coupled with lower circulating triglycerides—is consistent with improved hepatic lipid clearance and reduced steatosis risk. A key open question is whether these effects can be recapitulated non-surgically, through circulating ovarian peptides, endocrine factors, or small-molecule metabolic modulators. Identifying the specific ovarian-derived signals responsible will be an essential step toward therapeutic translation.

### Pharmacological and therapeutic connections

The metabolic changes observed in transplant recipients align with mechanisms targeted by several emerging therapeutic strategies. PPARα agonists (e.g., fibrates) regulate hepatic *β*-oxidation and lipid handling; the reduced expression of PPARα target genes in recipients may reflect an endogenous PPARα-like reprogramming. CPT1A and CPT2 expression changes suggest potential relevance of CPT1 modulators, which control the rate-limiting step of mitochondrial fatty acid import. Mitochondria-targeted therapeutics (e.g., MitoQ) are also pertinent given the improved oxidative stress profiles in recipients [52]. Finally, the metabolic phenotype of FC/FD mice shares features with NAD^+^/sirtuin-replete states—SIRT1 and SIRT3 regulate fatty acid oxidation and mitochondrial homeostasis in an NAD^+^-dependent manner that declines with age—raising the possibility that ovarian-derived signals converge on this pathway. Each of these connections represents a testable hypothesis and a potential avenue for pharmacological intervention.

## CONCLUSION

Transplantation of young ovarian somatic tissue is associated with comprehensive metabolic remodeling in aged mice, characterized by reduced *β*-oxidation protein expression and markedly lower triglyceride levels. These proteomic shifts are consistent with ovarian-derived factors supporting restoration of mitochondrial efficiency and oxidative balance [52], reflecting a shift toward a more youthful metabolic profile. These findings position the ovarian somatic environment as an important regulator of systemic metabolic health and a potentially compelling target for interventions aimed at extending health span in aging females.

## Figures and Tables

**Table 1.** Metabolic cage analysis

Metabolic cage data		
	Bursal transplant	Control
VolO_2_ (L min^−1^)	1.79	1.98
VolCO_2_ (L min^−1^)	2.38	2.51
RQ	0.75	0.79
Feed (g day^−1^)	3.05	2.09
Drink (mL day^−1^)	2.87	3.01

Oxygen consumption (VO_2_, L min^−1^), carbon dioxide production (VCO_2_, L min^−1^), respiratory quotient (RQ = VCO_2_/VO_2_), heat production, food intake, water intake, and ambulatory activity were measured using a Comprehensive Lab Animal Monitoring System (CLAMS). Measurements were recorded at 10-min intervals over a 48-h period following a 24-h acclimation.

RQ values provide an index of substrate utilization, with lower values (&ap;0.7) indicating greater fatty acid oxidation and higher values (&ap;1.0) indicating predominant carbohydrate oxidation. Data are presented as mean values across light and dark cycles. Abbreviations: VO_2_, oxygen consumption; VCO_2_, carbon dioxide production; RQ, respiratory quotient; CLAMS, Comprehensive Lab Animal Monitoring System.

**Table 2.** Body weight and feed intake in CBA/J mice over a 49-day monitoring period

Weight and feed intake data			
	Starting weight (g)	End weight (g)	Average Feed Intake (g)
13 mo CTL	27.67	26.54	28.88
27 mo CTL	24.86	21.54	32.21
FC	26.32	26.31	40.67
FD	27.73	26.65	37.18

The table presents starting and final body weights, as well as average weekly feed intake, for control and ovarian transplant groups. Experimental groups include 13-month control (13 mo CTL), 27-month control (27 mo CTL), follicle-containing ovarian graft recipients (FC), and follicle-depleted ovarian graft recipients (FD).

Abbreviations: CTL, control; FC, follicle-containing ovarian tissue; FD, follicle-depleted ovarian tissue.

## Data Availability

The datasets generated and analyzed during the current study, including protein expression values, triglyceride measurements, and metabolic cage outputs, are available from the corresponding author on reasonable request.
